# Liquid-based cytology for primary cervical cancer screening: a multi-centre study

**DOI:** 10.1054/bjoc.2000.1588

**Published:** 2001-02

**Authors:** J Monsonego, A Autillo-Touati, C Bergeron, R Dachez, J Liaras, J Saurel, L Zerat, P Chatelain, C Mottot

**Affiliations:** Institut Alfred Fournier, 25 boulevard Saint Jacques, Paris, 75014, France; Faculté de Médecine Nord, Laboratoire Bio-cellulaire, boulevard Pierre Dramard, Marseille, 13916, France; Laboratoires Pasteur Cerba, 95066 St. Ouen L'Aumône, France; Département de Pathologie, LCL – Laboratoires Claude Lévy, 78 avenue de Verdun, Ivry sur Seine, 94200, France; Laboratoire GRC, 1 rue Louis Guérin, Villeurbanne, 69626, France; Laboratoire I.H.C.P., Z.A. du Limancet, 114–116 avenue Léon Blum, Le Bouscat, 33495, France; Laboratoires Lavergne-Victor Hugo, 33 avenue Victor Hugo, Paris, 75116, France; Phoenix International France, 6 avenue de la Cristallerie, Sèvres, 92316, France; Centre Georges François Leclerc, 1 rue du Professeur Marion, Dijon, 21034, France

**Keywords:** cervical smears, CIN, cervical cancer, ThinPrep cytology, cancer screening

## Abstract

The aim of this six-centre, split-sample study was to compare ThinPrep fluid-based cytology to the conventional Papanicolaou smear. Six cytopathology laboratories and 35 gynaecologists participated. 5428 patients met the inclusion criteria (age > 18 years old, intact cervix, informed consent). Each cervical sample was used first to prepare a conventional Pap smear, then the sampling device was rinsed into a PreservCyt vial, and a ThinPrep slide was made. Screening of slide pairs was blinded (*n* = 5428). All non-negative concordant cases (*n* = 101), all non-concordant cases (*n* = 206), and a 5% random sample of concordant negative cases (*n* = 272) underwent review by one independent pathologist then by the panel of 6 investigators. Initial (blinded) screening results for ThinPrep and conventional smears were correlated. Initial diagnoses were correlated with consensus cytological diagnoses. Differences in disease detection were evaluated using McNemar's test. On initial screening, 29% more ASCUS cases and 39% more low-grade squamous intraepithelial lesions (LSIL) and more severe lesions (LSIL+) were detected on the ThinPrep slides than on the conventional smears (*P* = 0.001), including 50% more LSIL and 18% more high-grade SIL (HSIL). The ASCUS:SIL ratio was lower for the ThinPrep method (115:132 = 0.87:1) than for the conventional smear method (89:94 = 0.95:1). The same trend was observed for the ASCUS/AGUS:LSIL ratio. Independent and consensus review confirmed 145 LSIL+ diagnoses; of these, 18% more had been detected initially on the ThinPrep slides than on the conventional smears (*P* = 0.041). The ThinPrep Pap Test is more accurate than the conventional Pap test and has the potential to optimize the effectiveness of primary cervical cancer screening. © 2001 Cancer Research Campaign http://www.bjcancer.com


*British Journal of Cancer* (2001) **84**(3), 360–366
© 2001 Cancer Research Campaign
doi: 10.1054/ bjoc.2000.1588, available online at http://www.idealibrary.com on IDEAL®
http://www.bjcancer.com

### Liquid-based cytology for primary cervical cancer screening: a multi-centre study

**J Monsonego[1], A Autillo-Touati[2], C Bergeron[3], R Dachez[4], J Liaras[5], J Saurel[6], L Zerat[7], P Chatelain[8] and C Mottot[9]**

[1]Institut Alfred Fournier, 25 boulevard Saint Jacques, 75014 Paris, France; [2]Laboratoire Bio-cellulaire, Faculté de Médecine Nord, boulevard Pierre Dramard, 13916 Marseille, France; [3]Laboratoires Pasteur Cerba, 95066 St. Ouen L'Aumône, France; [4]LCL – Laboratoires Claude Lévy, Département de Pathologie, 78 avenue de Verdun, 94200 Ivry sur Seine, France; [5]Laboratoire GRC, 1 rue Louis Guérin, 69626 Villeurbanne, France; [6]Laboratoire I.H.C.P., Z.A. du Limancet, 114–116 avenue Léon Blum, 33495 Le Bouscat, France; [7]Laboratoires Lavergne-Victor Hugo, 33 avenue Victor Hugo, 75116 Paris, France; [8]Phoenix International France, 6 avenue de la Cristallerie, 92316 Sèvres, France; [9]Centre Georges François Leclerc, 1 rue du Professeur Marion, 21034 Dijon, France

**Summary** The aim of this six-centre, split-sample study was to compare ThinPrep fluid-based cytology to the conventional Papanicolaou smear. Six cytopathology laboratories and 35 gynaecologists participated. 5428 patients met the inclusion criteria (age > 18 years old, intact cervix, informed consent). Each cervical sample was used first to prepare a conventional Pap smear, then the sampling device was rinsed into a PreservCyt vial, and a ThinPrep slide was made. Screening of slide pairs was blinded ($n$ = 5428). All non-negative concordant cases ($n$ = 101), all non-concordant cases ($n$ = 206), and a 5% random sample of concordant negative cases ($n$ = 272) underwent review by one independent pathologist then by the panel of 6 investigators. Initial (blinded) screening results for ThinPrep and conventional smears were correlated. Initial diagnoses were correlated with consensus cytological diagnoses. Differences in disease detection were evaluated using McNemar's test. On initial screening, 29% more ASCUS cases and 39% more low-grade squamous intraepithelial lesions (LSIL) and more severe lesions (LSIL+) were detected on the ThinPrep slides than on the conventional smears ($P$ = 0.001), including 50% more LSIL and 18% more high-grade SIL (HSIL). The ASCUS:SIL ratio was lower for the ThinPrep method (115:132 = 0.87:1) than for the conventional smear method (89:94 = 0.95:1). The same trend was observed for the ASCUS/AGUS:LSIL ratio. Independent and consensus review confirmed 145 LSIL+ diagnoses; of these, 18% more had been detected initially on the ThinPrep slides than on the conventional smears ($P$ = 0.041). The ThinPrep Pap Test is more accurate than the conventional Pap test and has the potential to optimize the effectiveness of primary cervical cancer screening. © 2001 Cancer Research Campaign http://www.bjcancer.com

**Keywords**: cervical smears; CIN; cervical cancer; ThinPrep cytology; cancer screening

Invasive cancer of the uterine cervix is preventable when its precursor lesions are detected and treated early. Cervical cytology has been in use now for more than 50 years, and has proven itself to be the main weapon of defence against this disease (Koss, 1989). However, in order to effectively protect the population from cervical cancer, two keys elements must be in place – the maximum number of adult women must be reached with the screening test, and the quality and effectiveness of the test itself must be unquestionable.

The impact of cervical cytology screening has been demonstrated by steadily reduced rates of incidence and mortality from invasive cervical cancer in the developed countries over the last decades (Stenkvist et al, 1984; Weidmann et al, 1998). In some European countries, notably the Scandinavian countries, Great Britain and the Netherlands, population screening programmes have been organized. In others, such as France and the United States, cytological screening has become a part of regular preventative care, primarily by educating and motivating individual physicians and patients, with variations from country to country in the coverage of the population and the frequency of testing (Fender et al, 1998). In every country, increasing the participation of women – particularly in the older age groups – in cervical screening is a critical health policy goal (Fender et al, 1998; Sancho-Garnier, 1998).

Equally important as offering cervical screening to every woman is ensuring that the test that is used is as accurate as possible. In recent years, the accuracy of the conventional Pap smear has come under a great deal of scrutiny. A recent meta-analysis of the accuracy of the conventional Pap smear has reported widely varying false negative rates (AHCPR, 1999). Investigations into the sources of false negative errors have concluded that the majority are due to sampling errors, that is, no abnormal cells are found on the smeared slide upon review (Gay, 1985; AHCPR, 1999). Abnormal cells may also go undetected because of poor smear quality (Weintraub, 1997).

The liquid-based ThinPrep Pap Test was developed as a replacement to the conventional method of preparing the cervical cytological specimen, and was approved for clinical use in the United States in May, 1996. The sampling device(s) containing the cervical cell sample from the patient is rinsed directly into a vial containing PreservCyt (Cytyc Corporation, Boxborough, MA, USA), a buffered preservative fluid; the vial is then sent to the cytopathology laboratory for automated slide preparation using the ThinPrep 2000 Processor (Cytyc Corporation, Boxborough, MA, USA) (Linder and Zahniser, 1998).

In clinical trials and routine clinical practice, the ThinPrep Pap Test has been shown to be more effective than the conventional Pap smear in several ways including significantly improved detection of low-grade and high-grade intraepithelial lesions, and a significant improvement in specimen adequacy (Lee et al, 1997;

*Received 6 June 2000*
*Revised 18 September 2000*
*Accepted 18 October 2000*

*Correspondence to:* J Monsonego

Linder and Zahniser, 1997; Roberts et al, 1997; Bolick and Heuman, 1998; Corkill et al, 1998; Dupree et al, 1998; Papillo et al, 1998; Carpenter and Daveu, 1999; Diaz-Rosario and Kabawat, 1999; Guidos and Selvaggi, 1999; Wang et al, 1999; Yeoh et al, 1999; Weintraub and Morabia, 2000).

This study, conducted in France, is the first formal multi-laboratory, large-scale evaluation of the ThinPrep Pap Test in the European setting.

### METHODS

### Study organization

6 laboratories in France participated in the study, each laboratory obtaining cervical samples from 5 to 8 participating gynaecologists and their patients. A total of 35 gynaecologists participated in the study.

Before the study commenced, the 6 laboratory directors (4 cytopathologists and 2 cytologists) and their participating staff were trained to interpret ThinPrep slides, and also to use the Bethesda System for reporting the screening results (Kurman and Solomon, 1994). The study protocols and forms were reviewed and approved by the local Ethics Committee.

Patients were recruited sequentially in the participating gynaecologists' practices, from March 1998 to September 1998. According to the inclusion criteria, female patients aged 18 and older, attending regular cervical cancer screening, and who voluntarily gave their informed consent, were enrolled in the study. Patients were excluded from the study if they did not have a uterine cervix, or if the cervix could not be visualized by the clinician.

Of the total of 5782 patients enrolled, 354 patients had to be excluded from further analysis for logistical reasons: 338 patients because clinicians made two slides for the conventional smear, and 16 patients for other reasons including lost slides (6), having been entered twice (3), lacking a cervix (1) or being under 18 years of age (6). This left 5428 qualified patients for whom there was one conventional Pap smear and one ThinPrep slide for the initial screening, thus fulfilling the statistical goal of 900 patients per laboratory.

### Specimen collection and processing

At the patient's visit, the gynaecological examination was performed in the usual way. For the cervical sample, a broom-style collection device was used (Cervex Brush, Rovers B.V., Oss, The Netherlands). A conventional Pap smear was made first, then the remainder of the cellular material on the collection device was rinsed into a vial containing PreservCyt preservative fluid (Cytyc Corporation, Boxborough, MA, USA). The conventional Pap smear, the vial, and the patient paperwork were forwarded to the cytopathology laboratory where the ThinPrep 2000 device (Cytyc Corporation, Boxborough, MA, USA) was used to prepare a slide from the sample in the vial. This device automatically mixes the sample, extracts a controlled number of cells onto a disposable filter, and then transfers the cells to a glass slide.

The screening protocol at each laboratory was organized so that the conventional Pap smear and the ThinPrep slide from each patient were screened routinely but separately; the cytologist screening a slide was blinded to the diagnosis of the other slide from the same patient. Once the initial screening had been completed, the diagnoses obtained from the two slides for each patient were correlated by a contract research organization (Phoenix International France, Sèvres, France). All slide pairs with discrepant diagnoses ($n$ = 206), all concordant abnormal pairs (ASCUS and higher; $n$ = 101), plus a 5% random sample of the concordant normal cases ($n$ = 272) were sent to an independent cytopathologist (C.M.) for review, again using a blinded protocol. Discordant specimen adequacy was not used as a criteria for review. After the independent review, the 6 investigators worked as a panel to review the non-negative and non-concordant cases, as well as any cases that had been upgraded by the independent reviewer (total panel review $n$ = 335). The panel review diagnosis was determined by a majority decision.

The results of the initial screening, the independent review, and the panel review were tabulated and analysed by the contract research organization. Three diagnoses were recorded. The 'initial diagnosis' was the diagnosis from the first reading of each *slide* at the laboratory. The 'final diagnosis' for each *slide* was defined as the diagnosis from the last reading performed (initial, independent review or panel review) on that slide. The 'reference diagnosis' was determined for each *patient* by comparing the final diagnoses from both the ThinPrep and the conventional Pap slides and recording the most abnormal of the two diagnoses for that patient.

Following the initial screening, 52 cases were excluded from the data analysis because one or both of the slides was inadequate for evaluation, leaving 5376 slides for the analysis of the initial screening data. No attempt was made to make additional slides from the vials of unsatisfactory ThinPrep cases. The independent review determined one or both slides to be inadequate in 5 additional cases, leaving 5371 cases for the analysis of the reference diagnoses.

### Statistical analysis

For the statistical analysis, differences in the rates of disease detection between the two preparation methods were assessed statistically using McNemar's test (2-category data) and the Stewart-Maxwell test (3-category data).

### RESULTS

Table 1 shows selected characteristics of the 6 participating laboratories and their patient populations. The volume of smears processed annually by each laboratory varied from 4000 smears per year to 200 000 smears per year. The patient populations were fairly uniform across all 6 centres. The average age of patients was 41 years, and about 23% were post-menopausal.

Table 2 shows the results of the initial, blinded routine-screening of the conventional Pap smear and ThinPrep slide from each patient. 50% more cases of LSIL were detected on the ThinPrep slides ($n$ = 99) than on the conventional smears ($n$ = 66), and 18% more cases of HSIL (33 ThinPrep: 28 conventional smear HSILs). For LSIL, HSIL, and cancers combined, there was a statistically significant 39% increase in the detection of LSIL and more severe lesions with the ThinPrep method ($P < 0.001$).

In the initial screening, 29% more ASCUS cases were detected on the ThinPrep slides ($n$ = 115) than on the conventional Pap smears ($n$ = 89). The ASCUS:SIL ratio was lower for the ThinPrep

*© 2001 Cancer Research Campaign* 

**Table 1** Characteristics of the six participating laboratories and their patient populations

| Centre | Laboratory volume of Papanicolaou smears per year | Number of participating gynaecologists | Number of 'per protocol' patients[a] | Mean patient age (SD) | Post-menopausal patients (%) | Previous abnormal Papanicolaou smears (%) | Previous LSIL+ (%) | Abnormal this study LSIL + (%) |
|---|---|---|---|---|---|---|---|---|
| A | 200000 | 8 | 906 | 41 (12.8) | 23.1% | 8.7% | 5.2% | 2.0% |
| B | 20000 | 6 | 906 | 38 (11.8) | 16.4% | 11.3% | 8.6% | 2.1% |
| C | 56773 | 5 | 909 | 40 (12.8) | 22.7% | 14.2% | 5.9% | 1.2% |
| D | 100000 | 5 | 908 | 42 (12.7) | 25.4% | 8.8% | 7.3% | 1.8% |
| E | 4000 | 5 | 899 | 40 (12.6) | 22.9% | 13.1% | 5.1% | 1.1% |
| F | 70000 | 6 | 900 | 44 (13.2) | 28.2% | 13.8% | 10.6% | 1.2% |
| Total | N/A | 35 | 5428 | 41 (12.8) | 23.1% | 11.6% | 7.1% | 1.6% |

[a]All patients who meet the inclusion/ exclusion criteria, with available pair of slides. [b]Abnormal Papanicolaou smear in the patient's previous medical history. [c] Final diagnosis (after pathologist reviews) of LSIL+ on conventional Pap smear.

**Table 2** Results of initial screening, ThinPrep versus Conventional Pap test diagnoses, by Bathesda diagnosis categories[a]

| | | Conventional Pap test | | | | | | | |
|---|---|---|---|---|---|---|---|---|---|
| | | Negative | ASCUS | AGUS | LSIL | HSIL | SQ CA | GL CA | Total |
| | Negative | 5069 | 40 | 5 | 9 | 4 | 0 | 0 | 5127 |
| ThinPrep | ASCUS | 73 | 32 | 1 | 7 | 2 | 0 | 0 | 115 |
| Pap test | AGUS | 0 | 0 | 1 | 0 | 0 | 0 | 0 | 1 |
| | LSIL | 38 | 14 | 0 | 46 | 1 | 0 | 0 | 99 |
| | HSIL | 4 | 3 | 0 | 4 | 21 | 1 | 0 | 33 |
| | SQ CA | 0 | 0 | 0 | 0 | 0 | 0 | 0 | 0 |
| | GL CA | 0 | 0 | 0 | 0 | 0 | 0 | 1 | 1 |
| | Total | 5184 | 89 | 7 | 66 | 28 | 1 | 1 | 5376 |

[a]Bethesda category abbreviations used: ASCUS – Atypical squamous cells of undetermined significance. AGUS – Atypical glandular cells of undetermined significance. LSIL – Low-grade squamous intraepithelial lesion. HSIL – High-grade squamous intraepithelial lesion. SQ CA – Squamous cell carcinoma. GL CA – Glandular cell carcinoma.

method (115:132 = 0.87:1) than for the conventional Pap smear method (89:94 = 0.95:1); likewise, the ASCUS/AGUS:LSIL ratio was lower for the ThinPrep method (116:99 = 1.17) than for the conventional method (96:66 = 1.45).

In this study, the cytological diagnoses were verified by a two-stage pathologist review process, as described above. Pursuant to this review, there were 145 cases that were assigned a reference diagnosis of LSIL or higher (LSIL+). Table 3a shows the correlation of the two initial cytological diagnoses from the 145 cases with confirmed LSIL+ cytology. The ThinPrep initial diagnosis was LSIL+ in 69% of these cases (100/145); this was 18% higher than the proportion of conventional Pap smears originally diagnosed as LSIL+ (85/145 = 59%; $P = 0.041$). A similar analysis, this time for the 230 cases where the reference diagnosis was ASCUS and higher (ASCUS+), also showed a significantly higher rate of detection with the ThinPrep method (190/130 = 83%) than with the conventional smear method (151/230 = 66%; $P < 0.001$).

Specimen adequacy results from the initial screening are summarized in Table 4. The proportion of 'satisfactory (SAT)' slides was slightly higher for conventional Pap smear slides (91%) than for the ThinPrep slides (87%). The underlying reasons for which slides prepared by the two different methods were deemed to be 'unsatisfactory (UNSAT)' or 'satisfactory but limited by (SBLB)...' were distinctly different. There were more conventional smears than ThinPrep slides that were 'limited by' obscuring blood (110 vs. 3 cases), obscuring inflammation (37 vs. 11 cases), thickness of cells (20 vs. 0 cases), and air drying (10 vs. 1 cases). There were more ThinPrep slides than conventional smears that were 'limited by' a lack of endocervical component (642 vs. 315 cases).

### DISCUSSION

This study, the first formal multi-laboratory, large-scale evaluation of the ThinPrep fluid-based cervical cytological method in Europe, comes at a time when policies for women's health care and technological advances in cervical cytology are both developing rapidly in Europe. The goal of maximizing the participation of all adult women in cervical screening is being realized to varying degrees in different countries (Fender et al, 1998). In Europe, a screening frequency of about every 3 years is the standard (Stenkvist et al, 1984; Fender et al, 1998). Given the evidence that false negative rates for the conventional Pap smear are higher than they were once thought to be, a method that significantly increases the accuracy of the smear test at an incremental cost for a single test may be more cost effective than the alternative which is to screen every woman at more frequent intervals in order to catch the missed positive cases before they progress to cancer.

In this study, significantly more precancerous lesions were detected on slides prepared using the ThinPrep preparation method than on conventional Pap smear slides made first from the same cellular sample. 50% more LSIL lesions and 18% more HSIL lesions were detected in routine screening; taking LSIL and higher lesions together, there was a significant, 39% increase in detection with the ThinPrep method ($P < 0.001$). Numerous other recent

 © 2001 Cancer Research Campaign

**Table 3** Cases with non-negative reference diagnoses[a]: correlation of initial ThinPrep and conventional diagnoses

**a**: **Cases with reference diagnosis of LSIL and higher**: **ThinPrep versus conventional initial diagnoses**

| | | Conventional Pap test | | |
|---|---|---|---|---|
| | | **Negative/ASCUS/AGUS** | **LSIL+** | **Total** |
| ThinPrep | Negative/ASCUS/AGUS | 29 | 16 | 45 |
| Pap test | LSIL+ | 31 | 69 | 100 |
| | Total | 60 | 85 | 145 |

McNemar's test $P$ value = 0.041. Reference diagnosis LSIL+ cases initially detected with ThinPrep Pap test = 100/145 = 69.0%. Reference diagnosis LSIL+ cases initially detected with Conventional Pap test = 85/145 = 58.6%. Ratio of TP/CP confirmed positive initial diagnoses = 100/85 = 1.18. [a]Note: Reference diagnosis after expert/ panel review of all cases with non-negative initial diagnosis plus 5% of concordant negative cases.

**b**: **Cases with reference diagnosis of ASCUS/AGUS and higher**: **ThinPrep versus conventional initial diagnosis**

| | | Conventional Pap test | | |
|---|---|---|---|---|
| | | **Negative** | **ASCUS/AGUS+** | **Total** |
| ThinPrep | Negative | 13 | 27 | 40 |
| Pap test | ASCUS/AGUS+ | 66 | 124 | 190 |
| | Total | 79 | 151 | 230 |

McNemar's test $P$ value < 0.001. Reference diagnosis ASCUS+ cases initially detected with ThinPrep Pap test = 190/230 = 82.6%. Reference diagnosis ASCUS+ cases initially detected with Conventional Pap test = 151/230 = 65.7%. Ratio of TP/CP confirmed positive initial diagnoses = 190/151 = 1.26.

**Table 4** Specimen adequacy on initial diagnosis**:** comparison of conventional and ThinPrep slides

| **Specimen adequacy evaluation** | **Conventional** | | **ThinPrep** | |
|---|---|---|---|---|
| | **N** | **%** | **N** | **%** |
| Number of patients | 5428 | 100.00 | 5428 | 100.00 |
| Satisfactory for evaluation (SAT) | 4914 | 90.53 | 4726 | 87.07 |
| Satisfactory for evaluation but limited by (SBLB)**:** | 488 | 8.99 | 673 | 12.40 |
| Air drying artifact | 10 | 0.18 | 1 | 0.02 |
| Thick smear | 20 | 0.37 | 0 | 0.00 |
| Cylindrical endocervical cells or squamous metaplasia absent | 315 | 5.80 | 642 | 11.83[a] |
| Squamous epithelial cells scanly | 29 | 0.53 | 21 | 0.39 |
| Obscuring blood | 110 | 2.03 | 3 | 0.06[a] |
| Obscuring inflammation | 37 | 0.68 | 11 | 0.20[a] |
| No cells | 1 | 0.02 | 1 | 0.02 |
| Cytolysis | 6 | 0.11 | 3 | 0.06 |
| Other | 3 | 0.06 | 1 | 0.02 |
| Unsatisfactory for evaluation (UNSAT)**:** | 26 | 0.48 | 29 | 0.53 |
| Air drying artifact | 0 | 0.00 | 0 | 0.00 |
| Thick smear | 0 | 0.00 | 0 | 0.00 |
| Cylindrical endocervical cells or squamous metaplasia absent | 5 | 0.09 | 11 | 0.20 |
| Squamous epithelial cells scanty | 4 | 0.07 | 19 | 0.35 |
| Obscuring blood | 19 | 0.35 | 5 | 0.09 |
| Obscuring inflammation | 7 | 0.13 | 0 | 0.00 |
| No cells | 0 | 0.00 | 4 | 0.07 |
| Cytolysis | 2 | 0.04 | 1 | 0.02 |
| Other | 0 | 0.00 | 1 | 0.02 |

Stewart-Maxwell test for the 3 major categories of SAT, SBLB, UNSAT**:** $P$ < 0.001. [a]McNemar test, $P$ < 0.001. NOTE**:** Within the SBLB and UNSAT categories, a slide may have more than one factor.

studies concur unanimously that the ThinPrep method yields significantly higher detection of SIL lesions (Lee et al, 1997; Roberts et al, 1997; Bolick and Hellman, 1998; Corkill et al, 1998; Dupree et al, 1998; Papillo et al, 1998; Carpenter and Davey, 1999; Diaz-Rosario and Kabawat 1999; Guidos and Selvaggi, 1999; Wang et al, 1999; Yeoh et al, 1999; Weintraub and Morabia 2000).

Because this was the first multi-laboratory evaluation of the ThinPrep method in Europe, the study protocol was designed to be similar to that used for the clinical trial study in the USA, including the broom type collection device, a split sample from each subject, blinded screening, and independent pathologist review (Lee et al, 1997). The diagnosis results of this study were

*© 2001 Cancer Research Campaign* 

similar to those from the US clinical trial study: 18% increase in LSIL+ initial diagnoses ($P < 0.001$), confirmed by independent pathologist review.

Since this study was designed, the ThinPrep method has been widely adopted into routine clinical practice elsewhere, with the cervical sample collected directly to the vial, rather than split between two methods as was done in this study. There have been 4 studies in which cervical cytology from practices that have converted 100% to direct-to-vial, routine clinical use of the ThinPrep method have been compared to the performance of the conventional Pap smear method from the same physicians' practices one year prior to ThinPrep conversion (Dupree et al, 1998; Papillo et al, 1998; Carpenter and Davey, 1999; Diaz-Rosario and Kabawat, 1999). Taken together, these direct-to-vial studies showed more markedly improved screening results than this split-sample study did: significant average increases of 66% in LSIL detection ($P < 0.001$) and 57% in HSIL detection ($P < 0.001$), and a decrease in ASCUS diagnoses of 32% ($P < 0.001$).

In this study, the accuracy of the initial cytological screening was evaluated cytologically, using expert pathologist reviews. Since there was no way of assessing the competence of the 35 investigating gynaecologists in performing colposcopic biopsies, the accuracy of these examinations would have been difficult to interpret because of the expected variability of the results (Sellors et al, 1990; Hopman et al, 1995). For the 145 cases with reference (confirmed) diagnoses of LSIL and higher, there was an 18% higher rate of detection of LSIL+ on the initial diagnosis with the ThinPrep method ($P = 0.041$). Histological verification of the cytological diagnoses in patients diagnosed with abnormalities was not performed in this study. The reason is that for routine cervical screening populations, subjecting every patient with a non-negative diagnosis to colposcopy and biopsy would be neither practical nor cost-effective. There have however, been data published from four direct-to-vial studies in which cytology–histology correlation was made (Papillo et al, 1998; Carpenter and Davey, 1999; Diaz-Rosario and Kabawat, 1999; Guidos and Selvaggi, 1999). When the data are combined, the positive predictive value (PPV) of an LSIL diagnosis is found to be 76%, equal for both the ThinPrep method and the conventional smear method. For HSIL lesions, the PPV was 88% for ThinPrep and 90% for the conventional smear; for LSIL and HSIL together, the PPV was 80% for both methods (Papillo et al, 1998; Carpenter and Davey, 1999; Diaz-Rosario and Kabawat, 1999; Guidos and Selvaggi, 1999). This finding, that the positive predictive value of a SIL diagnosis is maintained when the ThinPrep method is used, means that the significant increases in SIL cytology diagnoses that have been documented for the ThinPrep method indicate a true increase in the detection of biopsy-confirmable disease.

In this study, 30% more cases of ASCUS were diagnosed with ThinPrep than on conventional smears. While in agreement with one other split-sample study (Corkill et al, 1998) and several studies in which different groups of physicians and patients were used for the test and control groups (Bolick and Hellman, 1998; Guidos and Selvaggi, 1999; Weintraub and Morabia, 2000), this finding is in contradiction with the US clinical trial study and most of the direct-to-vial studies published to date (Lee et al, 1997; Dupree et al, 1998; Papillo et al, 1998; Carpenter and Davey, 1999; Diaz-Rosario and Kabawat, 1999; Wang et al, 1999; Yeoh et al, 1999). It is difficult to interpret what this finding means. ASCUS is not a commonly used diagnostic category in the French cytology setting – both the Bethesda categorization and the slightly different visual characteristics of ThinPrep cytology were new to the cytologists and pathologists in this study. The laboratory directors received a one-week training in the use of the ThinPrep technique. With the exception of one centre which did have extensive experience with liquid-based cytology, none of the others had much, if any, experience with this method. In other ThinPrep studies, the number of ASCUS diagnoses has sometimes been found to increase (Bolick and Hellman, 1998; Corkill et al, 1998), and sometimes to decrease (Papillo et al, 1998; Wang et al, 1999), with the ThinPrep method. It is nevertheless accepted that with increasing experience with the ThinPrep method, the number of ASCUS cases decreases. At the same time, the ASCUS:LSIL ratio, an overall indicator of screening performance, was reduced with the ThinPrep method from 1.45;1 to 1.17:1. Both ratios were well within the range of good cytological practice, indicating that ASCUS was, not being over-diagnosed (Bolick and Hellman, 1998; Corkill et al, 1998; Dupree et al, 1998; Papillo et al, 1998; Carpenter and Davey, 1999; Diaz-Rosario and Kabawat, 1999; Guidos and Selvaggi, 1999; Wang et al, 1999; Yeoh et al, 1999; Weintraub and Morabia, 2000). The detection of ASCUS smears can however have an impact on public health. The evidence from biopsy review studies underscores the importance of ASCUS cytology in overall detection of high-grade lesions. Statistically, only 7% of individual patients with ASCUS cytology are found to have a high-grade lesion (Kinney et al, 1998) but looked at from the screening population, and because the prevalence of the ASCUS category of smears is higher than others (LSIL/HSIL), it has been shown that about 40% of histologically confirmed HSIL were preceded by an ASCUS cytological diagnosis (Kinney et al, 1998).

As has been found in other studies, the number of slides that were 'satisfactory but limited by...' obscuring blood, inflammation, thickness, and air drying, was much lower for the slides prepared by the ThinPrep method. In this study, there were more ThinPrep than conventional slides that lacked endocervical component. This result has been noted in other split-sample studies (Lee et al, 1997; Wang et al, 1999); the first portion of the sample was used to make the conventional smear and may have contained most of the endocervical component. Another factor may have been the necessity of introducing the 'broom' sampling device for this study. The spatula-endocervical brush combination might have yielded a higher proportion of samples with endocervical component. In every other published performance study of the ThinPrep 2000 device, particularly the direct-to-vial studies, there has been an improvement in smear adequacy – increased SAT cases and decreased SBLB cases – with the ThinPrep preparation method (Lee et al, 1997; Bolick and Hellman, 1998; Papillo et al, 1998; Carpenter and Davey, 1999; Diaz-Rosario and Kabawat, 1999; Guidos and Selvaggi, 1999; Wang et al, 1999; Yeoh et al, 1999; Weintraub and Morabia, 2000).

Cost-effectiveness studies of this new technology have been published, and are ongoing (AHCPR 1999; Brown and Garper, 1999; Hutchinson, 2000). In Great Britain, the National Institute for Clinical Excellence (NICE) of the National Health Service (NHS) has begun an appraisal of liquid-based cytology for cervical screening, which is scheduled to be published in 2000 (NICE, 2000a,b). The costs of the new technology will include the equipment cost of the ThinPrep 2000 device (at the laboratory), and the per test incremental cost of the disposable vial, the filter cylinder, and processing. Weighed against these will be the benefits of significantly increased screening accuracy and adequacy – less

 © 2001 Cancer Research Campaign patient recalls for inadequate or inconclusive sampling, and the maintenance of the 3-year screening interval due to significantly less missed positives. Higher rates of early disease detection will decrease the number of costly and severe treatments needed for late stage, invasive, or fatal disease.

Another potential benefit of the ThinPrep preparation method is that the remainder of the cell sample in the vial can be used for adjunctive testing (HPV and other diseases), without requiring the patient to be recalled (Sherman et al, 1997). HPV testing may be a promising way to more accurately recognize underlying significant disease in patients with atypical and low-grade cytology findings (Manos et al, 1999), and also in conjunction with cytology a useful screening tool.

In conclusion, this and other studies of the ThinPrep Pap Test show that this new technology significantly increases the detection of squamous intraepithelial lesions of the cervix. In routine use, when the entire sample is put into the vial, SIL detection and specimen adequacy are improved at higher rates than the results of this split-sample study. The ThinPrep Pap Test is strongly in the best interests of public health – by improving the quality of the sample and reducing the likelihood of false negative cytology results, it will significantly improve early detection and treatment of cervical abnormalities without dictating a change in screening intervals.

### CONTRIBUTORS

The study was independently organized and managed by a contract research organization, Phoenix International (Sèvres, France), specialists in the organization of clinical trials. The investigators were the directors of six public or private cytopathology laboratories in France which were selected for their expertise and independence as well as their representative role in the field of cytopathology in France. The patients were recruited and samples all taken by 35 co-investigator physicians. This study was supported by grants from Cytyc Corporation.

### ACKNOWLEDGEMENTS

We would like to extend grateful thanks to all the clinical collaborators: D Benmoura, J Champion, J-L Chiche, J Cohen, M Jourdan, Ch Lafond, D Leger, J-L Mergui, Ch Recours Nguyen, D Stolla, A Chabert, M-O Coste, C Decaudin, J Derrien, M-C Dulucq-Casalegno, C Guillet, Ch Lavagna-Maurice, F Lavieille, M Marien, Ch Paquet, S Vercambre, D Didi-Pige, N Fournier, N Galland, M Gaulier-Dessoit, I Heard, B Juan Bringuy, F Lévy, C Coulon, M Monfort, A Maarek, N Khebichat-Costa, G Robert, M-A Curtay-Rageul. We would also like to thank P Chatelain and A Rakotomanga (Phoenix International) who were responsible for the organization of the study and the data management, respectively.

### REFERENCES

Bolick DR and Hellman DJ (1998) Laboratory implementation and efficacy assessment of the ThinPrep cervical cancer screening system. *Acta Cytol* **42**: 209–213

Brown AD and Garber AM (1999) Cost-effectiveness of 3 methods to enhance the sensitivity of Papanicolaou testing. *JAMA* **281**: 347–353

Carpenter AB and Davey DD (1999) ThinPrep® Pap Test™: performance and biopsy follow-up in a university hospital. *Cancer (Cancer Cytopathol)* **87**: 105–112

Corkill M, Knapp D and Hutchinson ML 1998 Improved accuracy for cervical cytology with the ThinPrep method and the endocervical brush-spatula collection procedure. *Lower Genital Tract Disease* **2**: 12–16

Diaz-Rosario LA and Kabawat SE (1999) Performance of a fluid-based thin layer Pap smear method in the clinical setting of an independent laboratory and an out-patient screening population in New England. *Arch Pathol Lab Med* **123**: 817–821

Dupree WB, Suprun HZ, Beckwith DG, Shane JJ and Lucente V (1998) The promise and risk of a new technology: the Lehigh Valley Hospital's experience with liquid-based cervical cytology. *Cancer* **84**: 202–207

Evidence Report/Technology Assessment Number 5: Evaluation of Cervical Cytology (February 1999) Agency for Health Care Policy and Research. US Department of Health and Human Services. AHCPR Pub. No. 99–E010

Fender M, Schaffer P and Dellenbach P (1998) Peut-on et faut-il organiser le dépistage du cancer du col de l'utérus en France? [Is it possible and necessary to organise cervical cancer screening in France?]. *J Gynaecol Obstet Biol Reprod* **27**: 683–691

Gay J, Donaldson L and Goellner J (1985) False negative results in cervical cytologic studies. *Acta Cytol* **29**: 1043–1046

Guidos BJ and Selvaggi SM (1999) Use of the ThinPrep® Pap test™ in clinical practice. *Diagn Cytopathol* **20**: 70–73

Hopman EH, Voorhost FJ, Kenemans P, Meijer CJLM and Helmerhorst TJM (1995) Observer agreement on interpreting colposcopic images of CIN. *Gynecol Oncol* **58**: 206–209

Hutchinson ML, Berger BM and Farber FL (2000) Clinical and cost implications of new technologies for cervical cancer screening: the impact of test sensitivity. *Am J of Manag Care* **6**: 766–780

Kinney WK, Manos MM, Hurley LB and Ransley JE (1998) Where's the high-grade neoplasia? The importance of minimally abnormal Papanicolaou diagnoses. *Obstet Gynecol* **91**: 73–76

Koss LG (1989) The Papanicolaou test for cervical cancer detection: a triumph and a tragedy. *JAMA* **261**: 737–743

Kurman RJ and Solomon D (1994) *The Bethesda system for reporting cervical/vaginal cytologic diagnoses: definitions, criteria and explanatory notes for terminology and specimen adequacy*. Springer-Verlag: New York

Lee KR, Ashfaq R, Birdsong GG, Corkill ME, McIntosh KM and Inhorn SL (1997) Comparison of conventional Papanicolaou smears and a fluid-based, thin-layer system for cervical cancer screening. *Obstet Gynecol* **90**: 278–284

Linder J and Zahniser D (1997) The ThinPrep Pap Test: a review of clinical studies. *Acta Cytol* **41**: 30–38

Linder J and Zahniser D (1998) ThinPrep Papanicolaou testing to reduce false-negative cervical cytology. *Arch Pathol Lab Med* **122**: 139–144

Manos MM, Kinney WK, Hurley LB, et al (1999) Identifying women with cervical neoplasia using human papillomavirus DNA testing for equivocal Papanicolaou results. *JAMA* **281**: 1605–1610

National Institute for Clinical Excellence (2000) *First work programme for the National Institute for Clinical Excellence* (press release, 06 August 1999) [Online]. Available: http://www.nice.org.uk/updates/press/nice_pr.htm [2000, February 21]

National Institute for Clinical Excellence (2000) *NICE technology appraisals: programme of results announced* (press release issued 09 December 1999) [Online]. Available: http://www.nice.org.uk/updates/press/0912pr.htm [2000, February 21]

Papillo JL, Zarka MA and StJohn TK (1998) Evaluation of the ThinPrep Pap Test in clinical practice: a seven-month, 16,314-case experience in northern Vermont. *Acta Cytol* **42**: 203–208

Roberts JM, Gurley AM, Thurloe JK, Bowditch R and Laverty CRA (1997) Evaluation of the ThinPrep Pap Test as an adjunct to the conventional Pap smear. *Med J Aust* **167**: 466–469

Sancho-Garnier H (1998) Dépistage des cancers du col de l'utérus en France [Screening for cancers of the uterine cervix in France]. *Gynécologie-Obstétrique Pratique* **105**: 1

Sellors JW, Nieminen P, Vesterinen E and Paavonen J (1990) Observer variability in the scoring of colpophotographs. *Obstet Gynecol* **76**: 1006–1008

Sherman ME, Shiffman MH, Lorincz AT, et al (1997) Cervical specimens collected in liquid buffer are suitable for both cytologic screening and ancillary human papillomavirus testing. *Cancer (Cancer Cytopathology)* **81**: 89–97

Stenkvist B, Bergstrom R, Eklund G and Fox CH (1984) Papanicolaou smear screening and cervical cancer: what can you expect? *JAMA* **252**: 1423–1426

Wang T-Y, Chen H-S, Yang Y-C and Tsou M-C (1999) Comparison of fluid-based, thin-layer processing and conventional Papanicolaou methods for uterine cervical cytology. *J Formos Med Assoc* **98**: 500–505

*© 2001 Cancer Research Campaign*

Weidmann C, Schaffer P, Hedelin G, et al (1998) L'incidence du cancer du col de l'utérus régresse régulièrement en France [The incidence of cancer of the uterine cervix is regressing steadily in France]. *Bull Epid Hebd* **5**: 17–19

Weintraub J (1997) The coming revolution in cervical cytology: a pathologist's guide for the clinician. *Références en Gynécologie Obstétrique* **5**: 1–6

Weintraub J and Morabia A (2000) Efficacy of a liquid-based thin layer method for cervical cancer screening in a population with a low incidence of cervical cancer. *Diagn Cytopathol* **22**: 52–59

Yeoh GPS, Chan KW, Lauder I and Lam MB (1999) Evaluation of the ThinPrep Papanicolaou test in clinical practice: 6-month study of 16541 cases with histological correlation in 220 cases. *HKMJ* **5**: 233–239

 © 2001 Cancer Research Campaign
